# Neighborhood Design, Physical Activity, and Wellbeing: Applying the Walkability Model

**DOI:** 10.3390/ijerph14010076

**Published:** 2017-01-13

**Authors:** Adriana A. Zuniga-Teran, Barron J. Orr, Randy H. Gimblett, Nader V. Chalfoun, David P. Guertin, Stuart E. Marsh

**Affiliations:** 1 Udall Center for Studies in Public Policy, University of Arizona, Tucson, AZ 85719, USA; 2 Department of Ecology, University of Alicante, San Vicente del Raspeig 03690, Spain; barron.orr@gmail.com; 3 School of Natural Resources and the Environment, University of Arizona, Tucson, AZ 85721, USA; gimblett@ag.arizona.edu (R.H.G.); dpg@email.arizona.edu (D.P.G.); smarsh@email.arizona.edu (S.E.M.); 4 College of Architecture, Planning and Landscape Architecture, University of Arizona, Tucson, AZ 85719, USA; chalfoun@email.arizona.edu

**Keywords:** neighborhood design, walkability, physical activity, wellbeing

## Abstract

Neighborhood design affects lifestyle physical activity, and ultimately human wellbeing. There are, however, a limited number of studies that examine neighborhood design types. In this research, we examine four types of neighborhood designs: traditional development, suburban development, enclosed community, and cluster housing development, and assess their level of walkability and their effects on physical activity and wellbeing. We examine significant associations through a questionnaire (*n* = 486) distributed in Tucson, Arizona using the Walkability Model. Among the tested neighborhood design types, traditional development showed significant associations and the highest value for walkability, as well as for each of the two types of walking (recreation and transportation) representing physical activity. Suburban development showed significant associations and the highest mean values for mental health and wellbeing. Cluster housing showed significant associations and the highest mean value for social interactions with neighbors and for perceived safety from crime. Enclosed community did not obtain the highest means for any wellbeing benefit. The Walkability Model proved useful in identifying the walkability categories associated with physical activity and perceived crime. For example, the *experience* category was strongly and inversely associated with perceived crime. This study provides empirical evidence of the importance of including vegetation, particularly trees, throughout neighborhoods in order to increase physical activity and wellbeing. Likewise, the results suggest that regular maintenance is an important strategy to improve mental health and overall wellbeing in cities.

## 1. Introduction—Background Information

Research has shown that the built environment affects lifestyle physical activity, and therefore human health [1,2,3]. The influence of the built environment on physical activity is referred to as walkability and has been a popular topic of research in recent years. Studies in the domains of public health, community development, land planning, and transportation domains have examined the relationship between the built environment and physical activity [4]. The field of urban design, however, has received less attention, even though it is the discipline that indirectly determines the walkability of the built environment. Urban design combines planning subdivision ordinances, zoning regulations, engineering street standards, and other metrics, to determine the street layout and accessibility of neighborhoods, the interactions between buildings and public spaces, the provision of greenspace and distribution of trees along the streets, and in many cases, the pedestrian and cycling infrastructure. Urban design therefore has the potential to influence physical activity and wellbeing. However, related research has been primarily focused on a limited number of neighborhood design types and has thus far produced inconsistent results on the effects of urban design on physical activity [5,6,7,8,9].

The purpose of this study is to address this gap by examining the relationship between four types of neighborhood designs with respect to those urban design factors that have been identified as contributing to human and social health. Specifically, we explore walkability, physical activity, wellbeing, perceived crime, the effects of trees, and the social interactions with neighbors. The four neighborhood designs considered are: (1) traditional development; (2) suburban development; (3) enclosed communities; and (4) cluster housing.

### 1.1. Traditional Development

The neighborhood design type we label as traditional development is composed of older “traditional” homes, the majority of which were built in the U.S. during or before the 1940s when most people did not own a car [10]. This type of development declined after the 1950s with the mass adoption of cars and the flight of wealthy residents to the suburbs [10].

Traditional neighborhood design provides compact housing (single-family houses usually with front porches that allow street surveillance), retail and offices within walking distance (less than half a mile or a ten-minute walk) and in some cases a small park [10]. Traditional development, which usually follows a grid street network with short blocks, and a mixture of land uses (residential and commercial), is thought to encourage walking for transportation [7,10]. When cars were introduced, traditional developments incorporated garages placed off the streets, facing alleys or service lanes [11]. This neighborhood design is thought to provide a range of vitality levels: commercial streets provide the services that generate lively activity, while nearby residential streets remain quiet and tranquil [10].

### 1.2. Suburban Development

Suburban development is also referred to as conventional residential development [12] or “the suburbs”. Suburban development began in the U.S. in the late 1940s with the construction of Levittown in Long Island. This form of development spread rapidly though the support of the federal mortgage system [11] and a number of regulations that transformed all cities in the U.S. [13]. By the 1950s, suburban development had become not only the neighborhood design norm in the U.S. [13], but also in other countries (e.g., China [14], South Korea [15], Russia, Hungary, Bulgaria [16], Lithuania [17], the Czech Republic [18], Spain [19], Canada [20], and South Africa [21]).

Suburban development is mainly composed of single-family housing of a maximized lot-size where most of the land is converted into houses, yards, driveways, sidewalks, and streets [12]. This design usually follows a dendritic (or tree-like) street network of cul-de-sacs where the traffic is fed into a few arterials or “branches” [10]. Suburban development seeks contact with nature and all the benefits that come with it, such as recreational opportunities, air quality, and views [12]. This type of design does not combine land uses, which means that there are generally no commercial destinations or services nearby. This separation of land uses makes it much less feasible to walk or bike for utilitarian purposes, and make walking for transportation generally unfeasible. However, this type of design has been found to be conducive to recreational walking [6].

The homogeneous low density of suburban developments has led to city-scale problems such as traffic jams, social segregation, car-oriented societies and the extensive consumption of natural landscapes [12]. Many scholars believe that the fewer opportunities for social interaction characteristic of the suburbs has resulted in decreased neighborhood satisfaction [12]. In spite of these negative effects, suburban development is still central to new developments worldwide [10,12,22].

### 1.3. Enclosed Community

Enclosed communities are neighborhoods that are gated and/or fenced. Gated communities originated in the late 1950s when middle and upper-income homogeneous neighborhoods started to occupy large tracts of land [23]. The idea of preventing the general public from entering a certain neighborhood and prohibiting the use of the public space originated in the U.S. in Baltimore in a neighborhood adjacent to the John Hopkins Hospital, and was later copied in New York and other cities [23]. Since then the construction of enclosed communities has become an important trend in how cities are growing in the U.S. and other parts of the world [24]. Proliferation of this type of development started in the 1990s in the U.S., spreading to Latin America [25,26], and has become a trend in Europe [27]. A few enclosed communities have evolved to include not only a low number of high-income single-family houses, but also several apartment buildings and even entire “little towns” that accommodate a diversity of housing types and services [27].

Because they tend to have a limited number of entrances, enclosed communities are thought to lead to longer and less direct routes, which negatively affect the connectivity of the whole city [28]. The segregating effect of enclosed communities is thought to hamper the “freedom of the city”—or the possibility to go anywhere [23]. This effect is linked to privatism, or the exclusion of the general public, which is thought to reduce sense of community [24]. However, through the structure of private governance (or homeowners’ associations), enclosed communities may provide an experience of community [24].

Enclosed communities are thought to raise income inequalities; they are not only a response to a fear of crime, but can reinforce this fear through their security-oriented approach to exclusion and lack of diversity in household income [25,26]. It has been argued that the primary motive behind the proliferation of gated communities is a sense of fear; and it is projected that the market demand for this type of neighborhoods will increase [29].

### 1.4. Cluster Housing

This design approach consists of clustering houses together in order to preserve natural open space (or greenspace) [12,22]. As a land planning alternative to the urban sprawl caused by suburban development, it was originally proposed by Randall Arendt and Robert Yaro at the University of Massachusetts in the late 1980s, and later followed by other urban designers [12,22]. This design approach is a reaction to the seemingly unstoppable momentum that suburban development achieved in the 1950s and the perception of a vanishing countryside [30]. This form of development is possible when homeowners are shared-owners of the open space between clusters, which is maintained through homeowners’ associations [31]. A competitive market for cluster housing developments emerged in the 1970s and the best-selling were the ones with housing units mimicking a detached home that are attached in a multi-unit complex, known commonly as townhomes [31]. Today cluster housing involves groups of dwelling units (usually in the form of townhomes). Each of these clusters shares servicing structures (e.g., swimming pools, tennis courts, community centers) in order to preserve greenspace [12]. Variants of this type of neighborhood design include, for example, country clubs where the open space is transformed into golf courses or marinas where there is a waterfront [31].

Cluster housing maintains approximately the same number of houses that would be built under the suburban development design approach; the difference is that the houses are grouped together and the remaining land is preserved as greenspace. Cluster housing increases the “perceived density” because houses are built right next to one another (townhomes). This high-perceived density is thought to decrease the levels of neighborhood satisfaction because of the perception of close proximity to other residents [12]. However, having a view of nature and access to greenspace helps ameliorate the negative effects of increased perceived density and is important for neighborhood satisfaction [12]. Sense of community in cluster housing is related to the frequency of use of greenspace [12].

### 1.5. Walkable Neighborhoods

Broadly speaking, walkable neighborhoods are characterized by high residential and retail density and a diversity of land uses that provide destinations for walking. They are well connected, have beautiful sights, and are designed at a pedestrian scale by including pedestrian infrastructure [4,32,33,34]. In a seminal study, Cervero and Kockelman [32] identified routes (how direct is the distance between destinations) and the 3 Ds—density, diversity (land uses), and design (pedestrian-oriented)—as the dimensions that influence modes of transportation, including walking and biking. Since then, research has identified many more neighborhood design elements that influence physical activity. In order to capture these more extensive research findings on walkability, we developed a Walkability Framework that integrates findings and hypotheses from multiple relevant research domains (e.g., physical activity, land planning, transportation, thermal comfort, health, and the built environment) in order to help architects, and urban designers address walkability in the built environment [35]. This framework builds upon previous studies on walkability [7,33,36,37,38,39,40,41] and the neighborhood design elements related to walkability used in the Leadership for Energy and Environmental Design for Neighborhood Development (LEED-ND) certification system [28]. The framework divides the neighborhood design elements into nine categories: *connectivity*, *land-use*, *density*, *traffic safety*, *surveillance*, *parking*, *experience*, *greenspace*, and *community*. In our exploration of the utility of this approach in accurately measuring the interactions between the built environment and physical activity we found significant correlations between all of the walkability categories (built environment) and people walking (physical activity), and we called this the Walkability Model [42]. In this study we apply this model to measure walkability in four neighborhood design types.

While there is a consensus in urban design that walkable neighborhoods are associated with lifestyle physical activity, causal evidence is still limited, largely because it is very difficult to manipulate the independent variable—neighborhoods [4]. The few studies that have examined the relationship between neighborhood design, walkability, and physical activity usually compare suburban developments (considered to be low-walkable) with another neighborhood type with what is hypothesized as a more walkable design, with inconsistent results [5,6,7,8,9]. For example, Saelens et al. [7] compared traditional developments with suburban developments, finding higher levels of physical activity in traditional developments. Other studies compared suburban development with a new urbanist neighborhood, finding that the new urbanist neighborhood showed higher levels of physical activity [5,8]. By contrast, Wells and Yang [9] looked at a new urbanist neighborhood and a suburban development, finding no significant results for physical activity. Rodríguez et al. [6] found mixed evidence when comparing new urbanist neighborhoods with suburban developments with regards to physical activity.

### 1.6. Motivations for Walking

The built environment affects lifestyle physical activity and therefore plays an important role in human health [13]. It has been recognized that incorporating moderate types of physical activity (e.g., walking, biking) into daily routines leads to a healthy lifestyle. For adults, it is recommended to do at least 30 min of physical activity every day of the week in order to obtain significant health benefits [13].

In order to explore walkability in different neighborhood designs, it is important to distinguish between two different motivations for walking: walking for recreation and walking for transportation [39,43,44]. Walking for recreation refers to walking with the sole purpose of exercise and recreation, whereas walking for transportation refers to walking with the purpose of reaching a destination [43,44]. Each type of walking is influenced by different characteristics of the built environment [42,45]. Evidence of the relationship of the built environment and the two motivations for walking is still limited and inconsistent, where most studies link the built environment to walking for transportation but not for walking for recreation [4,6,43]; where land-use (proximity to services as destinations for walking), traffic safety (pedestrian and bicycle infrastructure), and safety from crime (or surveillance) are important correlates [45]. However, a study by Spinney et al. [44] found that most recreational walking is home-based. In a systematic literature review on health and nature, Hartig et al. [45] reported mixed findings on the link between greenness of the built environment and physical activity; where the negative associations are caused by longer routes in greener neighborhoods, increased car-ownership, and large availability of parking that affect walking for transportation [45].

### 1.7. Wellbeing

While physical activity is an important component of human wellbeing, human health encompasses more. The World Health Organization (WHO) defines human health as “a state of physical, mental and social well-being and not merely the absence of disease or infirmity” [46]. In this study, we consider wellbeing as the combination of the physical, mental, and social health. Physical inactivity and sedentary lifestyles have not only been linked to physical health problems, but also can contribute to the risk of mental health illnesses including depression and attention deficit hyperactivity disorder [47], and sleep disorders [48]. Symptoms for all these ills can be reduced by exposure to regular physical activity [47,48].

Social health is negatively influenced by sedentary lifestyles. Walking is thought to increase social interactions among neighbors that over time can lead to a sense of familiarity, respect, and trust [4]. Neighborhoods that maximize social interactions through their designs are thought to increase sense of community, which has been associated with reduced street crime, enhanced child supervision, and higher levels of reported happiness [49]. Another factor that may influence social interactions is familiarity, or time living in a neighborhood [4]. In addition, research has revealed links between social cohesion in neighborhoods and greenspace [45]. However, other studies on walkability and neighborhood sociability have shown inconsistent findings and point to other contributing factors beyond the urban form [4].

An important factor to consider when examining wellbeing in cities is the nature–health nexus, particularly the presence of urban trees [50]. Research has linked the presence of trees to reduced air pollution [51], reduced crime [52], and better mental health [50]. In terms of physical activity, walking in green areas has been linked to lower levels of anger [45], increased self-esteem, and better mood [53]. In addition to nature, wellbeing relies on the provision of a supportive environment, freedom of choice, personal security, social relationships, adequate employment and income, access to educational resources, and cultural identity [54]. Walkable neighborhoods may provide all of the above.

## 2. Materials and Methods

**Figure 1:**
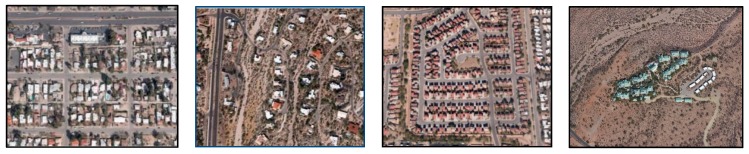
Four common neighborhood designs in Tucson, AZ, USA. From left to right, a traditional development, a suburban development, an enclosed community, and a cluster housing development. (Images from Google Earth).

In order to capture the perceptions, attitudes, and behavior of residents of the four neighborhood designs, we developed a questionnaire based on existing and validated tools [4,7,37,55,56] and questions adapted from LEED-ND [28]. A validation exercise allowed us to refine the questionnaire resulting in both an online and paper version [42]. The validated questionnaire was administered to residents of Tucson, Arizona between January and March 2014. Tucson was selected as the study site because it includes the four types of neighborhood designs of sufficient area within reasonably close proximity of each other, and of sufficient area to be captured in our sampling. Figure 1 shows the aerial photo of a prototype of each type of neighborhood design included in this study. Traditional development uses a grid street network with back alleys and has the proximity of services with homes. Suburban development involves single-family houses in a dendritic street network that is connected to an arterial road. Enclosed communities include restricted access points to the neighborhood through the use of fences/gates. The image for cluster housing depicts townhomes clustered together with shared facilities, while preserving greenspace.

The questionnaire has nine sections: (1) neighborhood design; (2) walkability; (3) physical activity; (4) wellbeing; (5) social interactions with neighbors; (6) familiarity; (7) perceived safety from crime; (8) trees; and (9) demographics. (See detailed questionnaire in [35,42]).

Neighborhood design was assessed with one main question: *Which picture most closely represents the street design of your neighborhood?* This is accompanied by the aerial images depicted in Figure 1. Previous tests determined that the technical terms used to describe these neighborhood design types (traditional, suburban, enclosed, cluster) were not always part of common usage. Where needed, the labeling of the design type was adjusted to match the commonly used term for that particular design type to ensure comprehension. The adapted and pre-tested legends employed are: (1) *Grid street network* for traditional development; (2) *Cul-de-sac streets* for suburban development; (3) *Enclosed community*; (4) *Clustered housing in the open desert* for cluster housing. To ensure we had achieved clarity in understanding of the four neighborhood design types, the questionnaire included a series of built-in validation questions of our categorization with the hypothesis that the characteristics captured in the questions and responses would correspond with the way respondents understood the neighborhood design types depicted in the images (Table A1 in Appendix A). The validation process used a Chi-square test to look for significant correlations between the way respondents replied to the characteristics of the neighborhood validation questions and the aerial image they selected.

The walkability section employs the Walkability Model [42], which was based on previously validated tools, including the Neighborhood Environment Walkability Scale (NEWS) [7,37], Walkability Index [38], and Walk Score [57]. To this we added design elements from studies on physical activity and urban planning [1,34], and from LEED-ND [28] (Table A2 in Appendix A). The *parking* category was not included in this study because there is ample parking availability throughout Tucson. All the walkability categories were added together and adjusted to a scale of 0 to 1 to derive the Walkability Index.

The physical activity section is divided into two parts: walking for transportation and walking for recreation. Both parts are based on the International Physical Activity Questionnaire (IPAQ) [55,58]. In addition, the section on social interactions with neighbors is based on Toit et al. [4] (Table A3 in Appendix A). The wellbeing section is composed of questions addressing physical, mental, and social health and was based on the 12-item Short Form Health Survey (12-SFHS) that measures self-reported physical and mental health [56]. For the social health component, we added two questions: *How much time during the past 4 weeks*… (i) *Have you had someone (or a pet) to walk with?* (ii) *Have you met with family and friends?* The 12th question from the 12-SFHS was moved to the social health component because it is focused on social activities. Familiarity is assessed with one question: *How long have you been living in your current neighborhood?* Perceived crime is assessed with one question: *Crime in my neighborhood makes it unsafe to go on walks* [37]. The presence of trees is assessed with one question: *Are there trees along the streets*? Demographics documented in the questionnaire include age, gender, race/ethnicity, income, and education. We used an even number in the Likert scale (4 points) in various questions in order to dichotomize the variable during the analyses (Strongly agree and Agree = Yes; Disagree and Strongly disagree = No). Most variables were normalized and converted into indices by adjusting the values to a scale of 0 to 1.

A web-based version of the questionnaire was distributed with the help of ward officials and neighborhood leaders who forwarded an invitation email with a link to the questionnaire to their listserv of residents. The majority of responses obtained through this online recruitment method came from residents of traditional developments (*n* = 189), followed by suburban developments (*n* = 40), enclosed communities (*n* = 17), and cluster housing (*n* = 3) (Table 1). Because we did not capture a well-balanced number of participants from the four neighborhood design types, we decided to recruit participants using two additional recruitment methods which required a paper-based version of the questionnaire. First, we recruited participants during several visits to the Rillito River Park, which is a greenway that has a walking/biking path along the Rillito River wash and extends for several miles. We chose this park because it is accessible to a gradient of socioeconomic populations and different types of neighborhoods in terms of design. Surveys were administered during the weekends of January and February of 2014. After these two methods of recruitment, our sample still included relatively few responses from the cluster housing design (*n* = 10) and the enclosed community design (*n* = 41). In order to increase the sample size of these two neighborhood designs, we decided to mail surveys to residents who were identified as living in these two types of neighborhoods in Tucson. A database was created with the addresses of houses in neighborhoods characterized by cluster housing and enclosed community designs. The resulting addresses were placed in a spreadsheet and then randomized; the first 150 entries were selected from this randomized list. We mailed surveys and collected responses during March 2014. The response rate of the mailed surveys was approximately 30% (*n* = 43).

Our recruitment method included people visiting a park, introducing a potential bias as they were already engaged in physical activity when recruited. To address this, we tested the random effect of the recruitment method variable (online, park, mail) with the dependent variables in a mixed model. By doing so, the effect of this variable was hopefully reduced and perhaps removed. Nevertheless, all the tests involving dependent variables in this study were adjusted for this random effect using the mixed model. The independent variables included neighborhood design (traditional, suburbs, enclosed, cluster), Walkability Index, and the walkability categories (*connectivity*, *density*, *land-use*, *traffic-safety*, *surveillance*, *experience*, *greenspace*, and *community*). Pairwise comparisons among the levels of the independent variables were conducted using the Bonferroni correction to adjust for alpha slippage in the post hoc analysis [59].

We also conducted a univariate analysis of variance (one-way ANOVA) to determine the magnitude of the relationships (R Squared), where we considered a moderate relationship when *R* was larger than 0.200. In addition, we performed bivariate correlations to test the relationship between non-categorical variables, and we considered moderate results when the Pearson correlation coefficient (*r*) was found larger than 0.30. Finally, we conducted Chi-square tests to validate our assessment of neighborhood design using the aerial images. The statistical analysis was performed using IBM-SPSS (IBM, Armonk, NY, USA) [60]. The Institutional Review Board of our academic institution approved this research for the protection of human subjects on 12 December 2013 (IRB # 13-0855 UAR Number 1300000855).

## 3. Results

Analysis of the demographics revealed 46.4% of the sample population to be over 60 years of age. In total, 63.1% were female, 87.7% white, 48.4% high-income, with 46.7% possessing a university/college degree (Table 2). With respect to neighborhood design type, we found significant relationship between age and design type (*p* < 0.001). Traditional developments showed a distribution where the highest age group corresponded to people in their 60s; suburban developments showed a quasi-homogenous distribution among people in their 50s and older; enclosed communities showed a peak in the age group in their 50s; and cluster housing has a prevalent population of people over 60 (Table 2). The highest number of young people (age group of 18–29) was reported in traditional developments (*n* = 12). The relationship between neighborhood design type and income was also found to be significant (*p* < 0.001). Most of the low-income respondents lived in traditional developments (*n* = 56) and less so in suburban developments (*n* = 10), while the other designs show very low numbers of low-income people within the sample population (*n* = 2, 1). The neighborhood design that showed the greatest diversity of income was traditional development. The other demographic variables (gender, education and ethnicity/race) were not found to be significant in relation to neighborhood design.

Our assessment of neighborhood design types through the use of the aerial pictures (Figure 1) provided significant results (*p* < 0.001) when tested with most of the hypothesized design characteristics using a Chi-square test. The only design characteristic that did not show significant associations with the aerial image was greenspace in close proximity (within 10-min walking distance; Table 3). Nevertheless, our hypothesis that cluster housing would obtain the highest percent of affirmative responses to having greenspace in close proximity was supported in this validation exercise. Likewise, our hypothesized characteristics for each neighborhood design type were confirmed. Our assessment of neighborhood design types and the estimated home age corresponds to the dates when the neighborhood design types became predominant in the U.S. The relationship between age of homes and neighborhood design types showed traditional development with a higher count for homes built on or before the 1950s. Suburban developments showed the highest percentage of homes built between the 1960s and 1980s followed by the 1950s and 1990s. Enclosed communities showed the highest percentage of homes built in the 1990s. Cluster housing shows a peak in the 1960s–1980s. Traditional developments showed the highest percentage of people reporting living in neighborhoods with back alleys, back alleys serving most of the garages, and front porches. Similarly, suburban developments showed the highest percentage of people reporting living in single-family homes and in a neighborhood with a cul-de-sac street network. Enclosed communities showed the highest percentage of people reporting living in gated and/or fenced neighborhoods. Finally, most people living in cluster housing reported living in townhomes, and sharing facilities.

Because our recruitment method included people visiting a park, we tested the random effect of the recruitment method variable (online, park, mail) with the dependent variables using a mixed model. The results are presented in Table 4.

Likewise, because our demographics showed a higher number of participants in their 60s or over, and mostly with high-income, we tested the random effects of these socio-demographic variables using our mixed model (Table 5).

**Figure 2:**
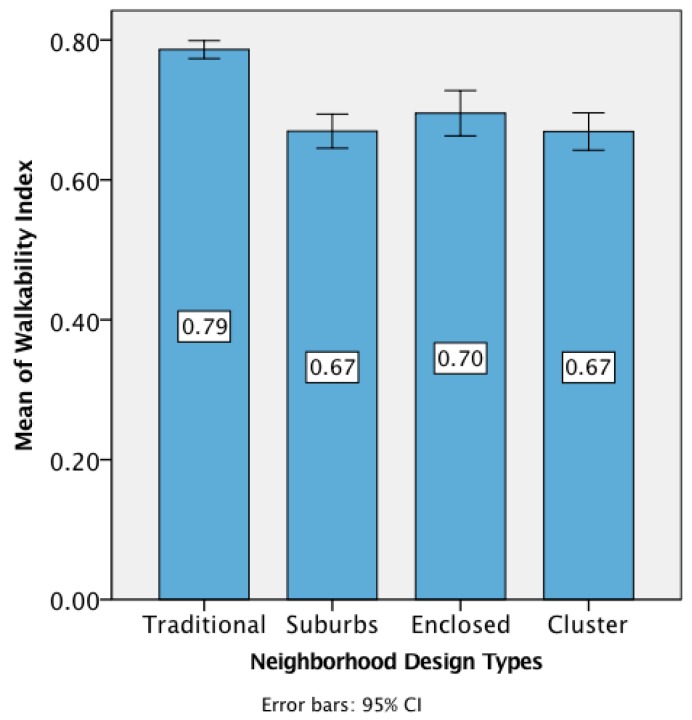
The relationship between neighborhood design and Walkability Index shows the highest mean for traditional development, significantly different from the other neighborhood design types in terms of walkability.

Our mixed model revealed that the only neighborhood design type that was significantly related to walkability was traditional development (*p* < 0.001). Traditional development showed the highest mean of the Walkability Index and its confidence intervals did not overlap with the confidence intervals of the other neighborhood design types (Figure 2).

We analyzed the relationship between each walkability category and neighborhood design type through a univariate analysis of variance (one-way ANOVA) and we found that all the walkability categories were significantly associated (Table 6). Traditional development obtained the highest mean for *connectivity*, *land-use*, *traffic safety*, *surveillance*, *greenspace*, and *community*. Cluster housing obtained the highest mean for *density* and *experience*. With regard to the magnitude of the relationships (*R*), neighborhood design was moderately related to the Walkability Index and several walkability categories including *connectivity*, *land-use*, *surveillance*, and *community*.

In order to measure self-reported physical activity with regards to neighborhood design type, we used our mixed model adjusting for the random effect of recruitment method and found a significant association between neighborhood design type and physical activity (Table 7). We also performed a one-way ANOVA and found a small magnitude in this relationship (*R* < 0.200). Likewise, when the two types of walking were considered (recreation and transportation), we found significant and small associations with neighborhood design type. Traditional development obtained the highest mean of physical activity, walking for recreation, and walking for transportation.

We examined the relationship between social interactions with neighbors and neighborhood design type using our mixed model and a one-way ANOVA, but we did not find significant results. However, when we performed the same analysis between social interactions with neighbors and walking for recreation we found significant (*p* = 0.012) and strong (*R* = 0.825) results. In addition, the relationship between social interactions with neighbors and walking for transportation showed a significant (*p* = 0.003) although small (*R* = 0.129) association. We tested familiarity—or time living in the neighborhood—and social interactions with neighbors and we obtained significant (*p* < 0.001) and strong (*R* = 0.648) results.

In order to test the relationship between the levels of self-reported wellbeing and its three components (physical, mental, and social health) with neighborhood design type, we used our mixed model and we found significant results for wellbeing and mental health (Table 8). We also conducted a one-way ANOVA to test the magnitude of these relationships and found small associations. Suburban development obtained the highest mean value for wellbeing and mental health according to neighborhood design type. With regard to the relationship between perceived safety from crime (the reversed values of perceived crime) and neighborhood design type, we found significant associations and cluster housing showed the highest mean value.

We explored the relationship between the Walkability Index (all the walkability categories together) and perceived crime using our mixed model and we did not find significant associations. However, we found significant results when considering the walkability categories individually, particularly *density*, *traffic safety*, and *experience* (Table 9). By conducting a one-way ANOVA, we tested the magnitude of the relationships and we found that the *R* values were small for *density*, and *traffic safety*. The relationship between the *experience* category and perceived crime was found to be moderately and inversely significant; as the values for the experience category increased the values for perceived crime decreased.

**Figure 3:**
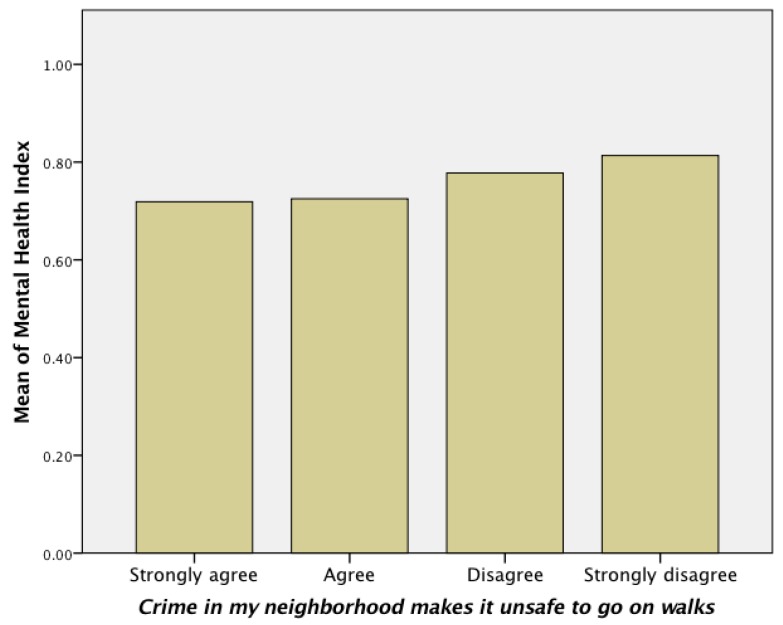
The relationship between perceived crime and mental health was found significant and inverse.

Our analysis revealed that neither physical activity nor the two motivations of walking (transportation and recreation) were significantly associated with perceived crime. However, perceived crime was significantly associated to wellbeing (*p* = 0.002; *R* = 0.092), and mental health (*p* = 0.001; *R* = 0.59), and these associations were inverse. For example, as the values of perceived crime decreased, the mean values of mental health increased (Figure 3). Because we found perceived crime significantly related to the presence of incivilities (trash, graffiti, and stray dogs; *p* < 0.001; *R* = 0.193), we examined the relationship between mental health and incivilities and found significant results (*p* = 0.028; *R* = 0.056).

Using our mixed model, we tested the relationships between presence of trees and other dependent variables; and we found significant results with physical activity, walking for recreation, social interaction with neighbors, perceived crime, and social health (Table 10). Moderate associations were found between presence of trees and walking for recreation, and strong associations between presence of trees and social interactions with neighbors.

Finally, we tested the relationships between physical activity and wellbeing using our mixed model and by conducting a bivariate correlation and we found significant results for wellbeing, physical health, and social health (Table 11). The magnitudes of the correlations, however, were weak (*r* < 0.3).

## 4. Discussion

### 4.1. Demographics

In a manner similar to several previous studies, the sample population in the present research demographically tended to be female [61,62], people of higher academic achievement [62,63], people of higher income [64], and people of white ethnicity [63]. However, our results in terms of race/ethnicity, age, education, and income are much higher than expected. This may be explained by local factors including the increase of retirees in Tucson during the winter season, when the data were collected, which could elevate the numbers of those who are white, older, with higher education, and with a higher income level [65]. Another possible explanation is the recruitment method. Web-based surveys may leave out participants with no access to a computer or to the internet [64], which may be related to income and education. Nevertheless, we tested the random effects of the sociodemographic variables that may have influenced health outcomes (age and income) and we did not find significant results.

With regard to the demographic distribution according to neighborhood design type, the high number of young participants living in traditional developments could be explained locally, by the proximity to the University of Arizona, where a high number of college students live. The high number of people 60 years and over in cluster housing corresponds to the era when this design became popular—the late 1980s—and when this cohort was in their 30s (a time and age cohort associated with buying homes to raise families). Likewise, the high number of people in their 50s who live in enclosed communities corresponds to the high proliferation of gated communities in the 1990s [25,26], when people in this age cohort were starting their families.

In terms of the income distribution according to neighborhood design type, we found that income inequalities were greater in the cluster housing and enclosed community designs. This is compatible with the findings of Le Goix and Webster [26], who linked gated communities to income inequalities. In the case of cluster housing, income inequality may be caused by the lack of diversity of housing types (most of the dwelling units are townhomes).

We consider that the assessment of neighborhood design type using the aerial image proved successful because our results supported all but one of our hypotheses of neighborhood design characteristics that define each type of design, the exception being the proximity and access to greenspace. We hypothesized that cluster housing would have a higher percent of responses for the *greenspace* category (proximity and access to greenspace or natural open space) and it did, but this variable was not significant to neighborhood design.

### 4.2. Walkability

Our analysis showed traditional development as the most walkable neighborhood design type, significantly different in terms of walkability from the other design types. Traditional development obtained the highest mean value for most walkability categories including: *connectivity*, *land-use*, *traffic safety*, *surveillance*, *greenspace*, *community*, and the Walkability Index. However, cluster housing obtained the highest mean value for *density* and *experience*. A high value for *experience* in cluster housing was expected because the proximity to natural open space (or greenspace)—one of the main characteristics of this design type—enhances aesthetics (natural sights) and the thermal comfort aspect of this category (shade). The high mean value of *density* for the cluster housing design may be explained because our assessment of density used questions that inquire about the dwelling type of the respondent and the most prevalent dwelling type within the neighborhood as perceived by the respondent (Table A1 in Appendix A). Most respondents from cluster housing reported living in a townhome because this is the predominant dwelling type in such design (Table 3). Although we originally expected to find that cluster housing would obtain the highest mean for *greenspace* (because this is one of the main characteristics of this design type), we were not surprised when this did not happen, because this was the only validation question that was not found to be significant in our assessment of neighborhood design using the aerial images in Figure 1 (Table 3).

Nevertheless, our results provide insights on the walkability strengths of these two design types. On the one hand, traditional development is strong in terms of walkability because its grid street network provides short and direct routes (*connectivity*), has commercial destinations close to homes (*land-use*), its infrastructure provides safety to pedestrians (*traffic safety*), front porches and small building setbacks allow people from inside the building to watch the streets (*surveillance*), and there are parks nearby (*greenspace*). On the other hand, cluster housing is somehow walkable because this design clusters houses together increasing the perception of *density*, while the shared natural open space improves the experience of walking by adding beautiful sights and enhancing thermal comfort (*experience*).

### 4.3. Motivations for Walking

Our results suggest that neighborhood design influences physical activity, where traditional development—the most walkable design in this study—showed the highest level of physical activity. We found significant associations between neighborhood design type and physical activity, and the two types of walking (recreation and transportation). Traditional development obtained the highest mean value for physical activity and for both types of walking, with a higher magnitude for walking for transportation. These results are consistent with Toit et al. [4], who found that the types of walking influence the relationship between the built environment and physical activity, being stronger in the case of walking for transportation. Our results, however, do not align with Rodriguez et al. [6], who found a correlation between suburban development and walking for recreation, because in this study, suburban development obtained the lowest mean value for walking for recreation.

### 4.4. Social Interactions with Neighbors

We found that neighborhood design, as a whole, may not affect whether neighbors interact or not, but the motivation for walking and familiarity may do so. We did not find a significant association between social interactions with neighbors and neighborhood design type. This result does not align with previous findings that relate cluster housing to increased sense of community through the use of outdoor shared areas [12], and a reduced sense of community in enclosed communities [24], and suburban developments [12]. However, we found that the type of walking (recreation vs. transportation) affects social interactions with neighbors. Walking for recreation was significantly and strongly associated with social interactions with neighbors. This suggests that people who go on walks within their neighborhood for exercise, dog-walking, or simple recreation are more likely to interact with neighbors than people who walk for the purpose to reach a destination. We also found that familiarity—or time living in the neighborhood—may affect social interaction through a significant and strong association, which is consistent with Toit et al. [4].

### 4.5. Wellbeing

Our results imply that neighborhood design may influence wellbeing, not precisely through walkability but through the presence of nature. We expected that traditional development would obtain the highest values for physical health and overall wellbeing because it was the most walkable and obtained the highest values for physical activity and the two types of walking. But we did not find significant results for the relationship between neighborhood design type and physical health. We found, however, significant associations between neighborhood design and wellbeing, and mental health; but traditional development did not show the highest mean value. Instead, suburban development showed the highest mean value for both, wellbeing and mental health. This finding is contrary to New Urbanist theories that claim that long automobile trips required by this type of neighborhood design type are linked to higher levels of stress and other mental ills [10]. This favorable mental health result for suburban development may relate to the presence of nature throughout the neighborhood, where big lots allow for more space for vegetation in yards, increasing the sense of nature throughout the neighborhood. Following suburban development in the mean values for mental health was cluster housing, which is characterized by preserved natural open space (or greenspace) providing nature close to homes. This result aligns with other studies that link mental health benefits of nature in urban settings [66,67,68].

Irrespective of neighborhood design type, past research suggests that physical activity is related to better health [1,2,3], and our results support this finding. We found significant correlations between physical activity and physical health, social health, and overall wellbeing. However, we did not find significant results between physical activity and mental health, which does not align with previous studies [69,70]. We tested the relationship between mental health and presence of incivilities (trash, graffiti, and stray dogs) and we found significant results. This suggests that perception of neighborhood, particularly presence of incivilities, may be an important variable for mental health outcomes, potentially as much or more than the previously documented link between physical activity and mental health [66,71].

### 4.6. Perceived Crime

Our analysis suggests that neighborhood design may play a role in perceived crime, but in an unexpected way. It was surprising to find that enclosed communities, which are supposed to provide safety from crime through the use of gates and fences, were not perceived as safer than the other neighborhood design types (Table 8). Enclosed communities showed a lower mean value for perceived safety from crime than suburban developments and cluster housing, where such gates and fences are not built into the designs. Cluster housing was the safest neighborhood design, based on the perceptions of the respondents; while traditional development was perceived as the least safe.

Although the overall Walkability Index was not significantly related to perceived crime, the individual walkability categories provided interesting insights. Significant associations were found between perceived crime and *density*, and *traffic safety* (Table 9). This is consistent with other studies that found that walkability and perceived crime are correlated [72]. Because traditional development showed the highest values for the walkability category *traffic safety*, it was not surprising to find that this design type was also perceived as the least safe.

Even though we expected to find *density* significantly associated with perceived crime, it was unexpected to find that cluster housing obtaining the highest level of perceived density *and* the lowest level of perceived crime. We believe that this incongruence between higher density and lower perceived crime in cluster housing may be a consequence of the design itself (where homes are clustered together surrounded by natural open space), that may have an isolating effect from the rest of the city. These results in cluster housing may reflect a sense of living in close proximity to well-known neighbors, but far from strangers. In addition, cluster housing obtained the highest mean values for the *experience* category (Table 6), which was significantly and moderately associated with perceived crime but in an inverse relationship. Therefore, it was expected to find that this design type was perceived as the safest. This result is consistent with other findings where vegetation—particularly trees—throughout the neighborhood (a component of the *experience* category) is related to lower crime rates [52,73].

The use of the Walkability Model that separates the neighborhood design elements into categories [42] proved useful in determining which aspects of walkability are related to perceived crime and which are not. In this case, *connectivity*, *land use*, *surveillance*, *greenspace*, and *community* were not significantly related to perceived crime. It was unexpected, however, to find that the two walkability categories that could be related to perceived crime such as *connectivity* (offers multiple routes and is open to the public) and *greenspace* (may foster criminal activities if large in scale [23]) were not significantly associated with perceived crime. Furthermore, we expected to find a significant and inverse relationship between *surveillance* and perceived crime because surveillance—or the ability to watch people on the streets from inside buildings through front porches, short building setbacks, lighting, and other design elements—is thought to increase the perception of safety from crime. But in this study we did not find significant associations between the *surveillance* category and perceived crime. More research is recommended.

In addition to the correlations with walkability, perceived crime was found to play role in wellbeing. We found significant and inverse associations between perceived crime and mental health and overall wellbeing. As the values of perceived crime increased, health values decreased. Our results suggest that the presence of incivilities may affect the perception of crime, which may adversely affect mental health and wellbeing.

### 4.7. Trees

This study provides empirical evidence that a pleasant experience for walking (*experience* category) that includes trees throughout the streets is linked to a higher perception of safety, a higher level of walking for recreation, increased social interactions with neighbors and better social health. Our results support earlier findings where the greenness of the built environment was related to walking for recreation, social interaction with neighbors [45] and safety from crime [52].

### 4.8. Limitations

An important caveat in this study is that negative social interactions (conflicts among neighbors) were not assessed, though these may hinder wellbeing. Also, our assessment of perceived residential density did not capture the overall density of the neighborhood that includes greenspace. In addition, we did not include the walkability category *parking*. Furthermore, we had wide sample size differences across neighborhood design types. Moreover, while this study provides correlation, it does not provide causation. Finally, with the nature of cross-sectional studies like this one, there is always the possibility that people sort themselves out by willingness to engage in physical activity. People who like to walk may choose to live in a walkable neighborhood, whereas people who prefer to drive may choose to live in a non-walkable neighborhood.

## 5. Conclusions

To our knowledge, this is the first study that examines four types of neighborhood designs including enclosed community and cluster housing and their relationships to walkability, physical activity, and wellbeing. Moreover, the inclusion of enclosed community and cluster housing represents a new contribution. In this study we found that neighborhood design may play a role in the wellbeing of residents, with significant results. Through different levels of walkability, we found that neighborhood design may affect physical activity and the two motivations for walking, as well as perceived crime, mental health, and overall wellbeing.

Our analysis suggests that most design types provide some type of wellbeing benefit. We found that traditional development is the only neighborhood design type that is distinctively different from the others in terms of walkability, and is the most walkable. This design type also showed the highest levels of physical activity, including the two motivations for walking (recreation and transportation). However, traditional development also showed the highest levels of perceived crime.

Even though cluster housing was not the most walkable design type, it still showed wellbeing benefits. This design obtained the highest value for social interactions with neighbors and it was perceived as the safest from crime. Cluster housing showed the highest mean value for the *experience* category of walkability, which suggests that the distinctive features of cluster housing—clustered townhomes surrounded by natural open space (part of the *experience* category)—may play a role in social interactions with neighbors and perceived safety from crime.

Suburban development showed the highest mean value for mental health and overall wellbeing. These results may be related to the large size of the lots that likely include vegetation, particularly trees. These health benefits of suburban development may contribute to understanding why this type of design is still guiding development worldwide [10,12,22].

In this study we did not find outstanding wellbeing benefits in the enclosed community design type, including perceived safety from crime, a quality that is commonly assumed to be a reason for living in this type of community. With the recent proliferation of enclosed communities, it becomes important to continue doing research on both the perceptions of and actual safety benefits of this type of design, particularly because enclosed communities disturb the connectivity of the city as a whole [28].

The use of the Walkability Model [42] allowed us to identify which aspects of neighborhood design are related to physical activity. In the case of traditional development, it was the most walkable because it obtained the highest mean values of *connectivity*, *land-use*, *traffic safety*, *surveillance*, *greenspace*, and *community*, all with significant results. The use of the model also allowed us to identify the walkability categories related to perceived crime (*density*, and *traffic safety*), the category that was inversely related (*experience*), and the ones that were not related (*connectivity*, *land-use*, *surveillance*, *greenspace*, and *community*).

An important take-away message is that the presence of nature, particularly trees, may provide various benefits including walking for recreation, wellbeing, perceived safety from crime, and social interactions with neighbors. This study provides empirical evidence of the need to include vegetation, particularly trees, throughout neighborhoods in order to increase physical activity and wellbeing. Likewise, our study supports the idea that regular maintenance that removes or reduces the impact of incivilities is an important strategy to improve mental health and overall wellbeing. We conclude that regardless of neighborhood design type, enhancing nature in the neighborhood and having regular maintenance may improve wellbeing. More research is recommended on the effects of these two strategies (increasing nature and maintenance) in traditional development (the most walkable design) and their impacts on wellbeing, social interaction with neighbors, and perceived crime. These key enhancements to the most walkable design type may result in healthier outcomes.

Results from this study shed light on the links between different aspects of the built environment (through the walkability categories), and wellbeing for well-established patterns of development (four neighborhood design types). Increasing our understanding of the effects of neighborhood design can lead to healthier communities.

## Figures and Tables

**Table 1:** Count and percent of responses according to neighborhood design type and recruitment method. Percentages are presented in parenthesis.

Recruitment Method	Traditional	Suburbs	Enclosed	Cluster	Total
Online	189 (87.5%)	40 (54.8%)	17 (37.0%)	3 (6.7%)	249 (65.5%)
Park	26 (12.0%)	31 (42.5%)	24 (52.2%)	7 (15.6%)	88 (23.2%)
Mail	1 (0.5%)	2 (2.7%)	5 (10.9%)	35 (77.8%)	43 (11.3%)
Total	216 (100.0%)	73 (100.0%)	46 (100.0%)	45 (100.0%)	380 (100.0%)

**Table 2:** Count of demographics of the sample population by neighborhood design. Percentages of demographic cohorts within each neighborhood design type (columns) are presented in parenthesis.

Demographic Variables	Cohorts	Traditional	Suburbs	Enclosed	Cluster	Total
Age	18–29	12 (5.8)	3 (4.3)	3 (6.5)	2 (4.4)	20 (5.5)
	30–39	21 (10.2)	4 (5.8)	7 (15.2)	0 (0.0)	32 (8.7)
	40–49	38 (18.4)	10 (14.5)	6 (13.0)	2 (3.6)	56 (15.3)
	50–59	46 (22.3)	18 (26.1)	18 (39.1)	6 (13.3)	88 (24.0)
	60–69	62 (30.1)	15 (21.7)	6 (13.0)	17 (37.8)	100 (27.3)
	70 or more	27 (13.1)	19 (27.5)	6 (13.0)	18 (40.0)	70 (19.1)
	Total	206 (100.0)	69 (100.0)	46 (100.0)	45 (100.0)	366 (100.0)
Gender	Male	76 (37.6)	22 (33.3)	15 (32.6)	19 (43.2)	132 (36.9)
	Female	126 (62.4)	44 (66.7)	31 (67.4)	25 (56.8)	226 (63.1)
	Total	202 (100.0)	66 (100.0)	46 (100.0)	44 (100.0)	358 (100.0)
Race/ethnicity	Native American	3 (1.5)	0 (0.0)	1 (2.2)	0 (0.0)	4 (1.1)
	Asian	1 (0.5)	1 (1.5)	1 (2.2)	2 (4.8)	5 (1.4)
	Hispanic	14 (7.1)	9 (13.4)	6 (13.3)	1 (2.4)	30 (8.6)
	White	176 (89.8)	57 (85.1)	35 (77.8)	39 (92.9)	307 (87.7)
	2 or more	2 (1.0)	0 (0.0)	2 (4.4)	0 (0.0)	4 (1.1)
	Total	196 (100.0)	67 (100.0)	45 (100.0)	42 (100.0)	350 (100.0)
Income (in U.S. dollars)	$30,000 or less	56 (28.4)	10 (16.1)	2 (4.4)	1 (2.4)	69 (20.0)
	$30,001 to $59,000	65 (33.0)	18 (29.0)	16 (35.6)	10 (24.4)	109 (31.6)
	$60,000 or more	76 (38.6)	34 (54.8)	27 (60.0)	30 (73.2)	167 (48.4)
	Total	197 (100.0)	62 (100.0)	45 (100.0)	41 (100.0)	345 (100.0)
Education	High School	14 (6.9)	5 (7.4)	1 (2.2)	0 (0.0)	20 (5.5)
	Professional School	8 (3.9)	3 (4.4)	3 (6.5)	2 (4.4)	16 (4.4)
	University/College	91 (44.8)	32 (47.1)	24 (52.2)	21 (46.7)	169 (46.7)
	Master’s /PhD	90 (44.3)	28 (41.2)	18 (39.1)	21 (46.7)	157 (43.4)

**Table 3:** Results of Chi-square test between neighborhood design and validation questions and percentage of responses according to neighborhood design type. Shown in bold are significant results (*p* < 0.05) and highest percentages of affirmative answers.

Neighborhood Design Types	Questions	*p* Value	Answer Options	Traditional (%)	Suburbs (%)	Enclosed (%)	Cluster (%)
All designs	Age of home	**<0.001**	On or before 1950s	**66.8**	31.4	8.7	2.2
			Between 1960s and 1980s	19.2	45.7	37.0	**80.4**
			On 1990s or later	13.5	21.4	**52.2**	9.5
			Don’t know	0.5	1.4	2.2	2.2
Traditional development	Neighborhood has alleys	**<0.001**	Yes	**52.7**	15.7	4.3	8.7
			No	40.3	84.3	95.7	91.3
	Back alleys serve most garages	**<0.001**	Yes	**31.7**	4.3	6.5	6.5
			No	168.3	95.7	14.8	93.5
	Most dwellings have front porches	**<0.001**	Yes	**66.2**	44.3	46.7	33.3
			No	33.8	55.7	53.3	66.7
Suburban development	How common are detached single-family	**<0.001**	None	0.5	2.7	10.9	13.3
			A few	2.3	1.4	6.5	37.8
			Some	11.2	9.6	15.2	24.4
			Most	68.4	26.0	21.7	15.6
			All	35.5	**60.3**	45.7	8.9
	Neighborhood has many cul-de-sacs	**<0.001**	Yes	12.7	**87.1**	81.8	59.1
			No	87.3	12.9	18.2	40.9
Enclosed community	Gates	**<0.001**	Yes	2.4	7.4	**37.0**	4.4
			No	97.6	92.6	63.0	95.6
	Fences	**<0.001**	Yes	4.8	16.2	**55.6**	9.1
			No	95.2	83.8	44.4	90.9
Cluster housing	How common are townhouses	**<0.001**	None	32.2	56.3	45.7	6.7
			A few	36.6	14.1	10.9	8.9
			Some	27.3	21.1	26.1	15.6
			Most	3.4	8.5	13.0	**46.7**
			All	0.5	0.0	4.3	**22.2**
	Shared facilities	**<0.001**	Yes	19.1	35.3	44.4	**77.8**
			No	80.9	64.7	55.6	22.2
	Greenspace in close proximity	0.513	Yes	70.4	67.1	65.2	**78.3**
			No	29.6	32.9	34.8	21.7

**Table 4:** Results of mixed models testing the random effect of recruitment method in the dependent variables.

Dependent Variables with Potential Bias	Significance	Variance (%)
Physical activity	0.456	4.92
Walking for recreation	0.593	6.36
Walking for transportation	0.409	6.70
Wellbeing	0.774	0.47
Physical health	0.523	3.22
Mental health	0.606	1.36
Social health ^a^	-	0.00
Perceived crime	0.365	11.73
Social interaction with neighbors	0.366	12.74

^a^ Variance is too small to be estimated.

**Table 5:** Results of mixed models testing the random effect of sociodemographic variables in the dependent variables.

Sociodemographic Variables with Potential Bias	Dependent Variable	Significance	Variance (%)
Age	Physical activity	0.244	4.56
	● Walking for recreation	0.231	4.93
	● Walking for transportation	0.662	0.69
	Wellbeing	0.302	3.47
	● Physical health	0.172	10.88
	● Mental health	0.445	3.08
	● Social health	0.503	0.15
	Perceived crime	0.434	1.69
	Social interaction with neighbors	0.201	13.25
Income	Physical activity	0.644	7.6
	● Walking for recreation	0.534	1.36
	● Walking for transportation	0.797	0.50
	Wellbeing	0.560	1.68
	● Physical health	0.609	1.02
	● Mental health	0.800	0.39
	● Social health	0.704	0.85
	Perceived crime	0.441	3.24
	Social interaction with neighbors	0.440	2.62

**Table 6:** Univariate analysis of variance between the walkability categories and neighborhood design type. Shown in bold are the significant (*p* < 0.05), at least moderate (*R* > 0.200) results, and the highest mean value according to neighborhood design type.

Walkability Categories	*R*	*p*	Traditional	Suburbs	Enclosed	Cluster
*Connectivity*	**0.377**	**<0.001**	**0.79**	0.65	0.60	0.67
*Density*	0.130	**<0.001**	0.50	0.42	0.51	**0.67**
*Land-use*	**0.277**	**<0.001**	**0.56**	0.37	0.39	0.15
*Traffic safety*	0.142	**<0.001**	**0.65**	0.52	0.62	0.57
*Surveillance*	**0.283**	**<0.001**	**0.69**	0.52	0.55	0.53
*Experience*	0.113	**<0.001**	0.68	0.74	0.74	**0.78**
*Greenspace*	0.024	**0.028**	**0.76**	0.69	0.71	0.68
*Community*	**0.206**	**<0.001**	**0.73**	0.65	0.62	0.44
Walkability Index *	**0.252**	**<0.001**	**0.79**	0.67	0.71	0.67

* Values for the eight walkability categories added together and adjusted to a scale of 0 to 1.

**Table 7:** Results of a mixed model (*p*) and one-way ANOVA (*R*) between neighborhood design and physical activity and the two types of walking. Shown in bold are significant results (*p* < 0.05) and the highest mean values for each dependent variable.

Dependent Variables	*R*	*p*	Traditional	Suburbs	Enclosed	Cluster
Physical activity	0.052	**0.019**	**0.521**	0.416	0.456	0.434
Walking for recreation	0.023	**0.049**	**0.508**	0.400	0.479	0.466
Walking for transportation	0.088	**<0.001**	**0.464**	0.390	0.348	0.362

**Table 8:** Results of a mixed model (*p*) and one-way ANOVA (*R*) between neighborhood design and wellbeing and its three components (physical, mental, and social health) and perceived safety from crime. Shown in bold are significant results (*p* < 0.05) and the highest mean values.

Dependent Variables	*R*	*p*	Traditional	Suburbs	Enclosed	Cluster
Wellbeing	0.023	**0.045**	0.822	**0.859**	0.839	0.832
Physical Health	0.009	0.677	0.898	0.919	0.916	0.894
Mental Health	0.058	**<0.001**	0.764	**0.830**	0.779	0.822
Social Health	0.005	0.601	0.800	0.808	0.812	0.775
Perceived safety from crime	0.125	**<0.001**	3.085	3.431	3.333	**3.733**

**Table 9:** Results of a mixed model (*p*) and one-way ANOVA (*R*) between the walkability categories and perceived crime. Shown in bold are significant (*p* < 0.05) and at least moderate (*R* > 0.200) results.

Walkability Category Tested with Perceived Crime	*R*	*p*
*Connectivity*	0.069	0.264
*Density*	0.049	**0.025**
*Land-use*	0.146	0.209
*Traffic safety*	0.087	**0.041**
*Surveillance*	0.087	0.060
*Experience*	**0.255**	**<0.001**
*Greenspace*	0.012	0.183
*Community*	0.057	0.541
Walkability Index *	**0.210**	0.369

* Values for the eight walkability categories added together and adjusted to a scale of 0 to 1.

**Table 10:** Results of a mixed model (*p*) and one-way ANOVA (*R*) between presence of trees and other dependent variables. Shown in bold are significant (*p* < 0.05) and at least moderate results (*R* > 0.200).

Variables Tested with Perceived Presence of Trees	*R*	*p*
Physical activity	0.056	**0.001**
Walking for transportation	0.006	0.743
Walking for recreation	**0.376**	**<0.001**
Social interactions with neighbors	**0.674**	**<0.001**
Perceived crime	0.051	**0.046**
Physical Health Index	0.009	0.448
Mental Health Index	0.018	0.100
Social Health Index	0.026	**0.043**
Wellbeing Index	0.020	0.093

**Table 11:** Results of our mixed model (*p*) and bivariate correlations (*r*) between physical activity and wellbeing, and its three components. Shown in bold are significant (*p* < 0.05) or at least moderate (*r* > 0.3) results.

Variables	*r*	*p*
Wellbeing	0.205	**<0.001**
Physical Health	0.184	**0.001**
Mental Health	0.022	0.552
Social Health	0.260	**< 0.001**

## Appendix A

**Table A1:** Characteristics that describe the neighborhood design types considered in this study and the questions used for validation.

Neighborhood Design	Characteristics	Questions from Questionnaire Used for Validation
All	Traditional—Mostly 1950s or earlier	1. Estimate the decade your home was built. Options: 1950s or earlier, 1960s to 1980s, 1990s to present, Don’t know
	Suburban—Mostly after 1950s	
	Enclosed—Mostly 1990s or later	
	Cluster—Mostly 1960s and later	
Traditional development	Back alleys	2. Back alleys serve most of the garages in my neighborhood. 4 Pt. Likert Scale (4LS—Strongly agree, Agree, Disagree, Strongly disagree)
	Garages face back alleys	3. *My neighborhood has back alleys with garages* (4LS)
	Front porches	4. *Most dwellings have front porches.* (4LS)
Suburban development	Mostly single-family housing	5. How common are single-family houses in your neighborhood? Options: None, A few, Some, Most, All)
	Mostly cul-de-sacs street network	6. *The streets in my neighborhood have many cul-de-sacs.* (4LS)
Enclosed community	Gated	7. *My neighborhood is a gated community.* (4LS)
	Fenced	8. *My neighborhood is fenced in the outer boundary.* (4LS)
Cluster housing	Mostly townhomes	9. How common are townhomes in your neighborhood? Options: None, A few, Some, Most, All
	Shared facilities	10. *My neighborhood shares facilities (e.g., pool, tennis courts, community center)* (4LS)
	Greenspace in close proximity	11. Check the services that are located within 10 min walking distance (1/2 mile) or less from your home Option: Greenspace

**Table A2:** Inputs for the walkability categories from the questionnaire.

Walkability Category	Questions	Answer Options
Connectivity	*There are major barriers for walking*	4 Pt. Likert Scale Strongly agree Agree Disagree Strongly disagree
	1. *The distance between intersections is usually short (100 yards) **	
	2. *There are many alternative routes for getting from place to place **	
	3. *My neighborhood is a gated community*	
	4. *My neighborhood is fenced on the outer boundary*	
	5. *The streets in my neighborhood have many cul-de-sacs*	
	6. *Back alleys serve most of the garages in my neighborhood **	
Density	1. *Select the option that best describes your dwelling type unit*	Single family Townhome Apartment Multi-family Temporary home
	2. *How common are single-family housing? **	None A few Some Most All
	3. *How common are townhomes?*	
	4. *How common are apartments/condos? **	
Land-use	*Check the services that are located within 10 min walking distance (1/2 mile or less from your home (check all that apply)* Bus stop, Gym, Post office, Bank, Supermarket, Hair salon/barber, School, Police station, Food store with produce, Laundry/dry cleaner, Theater, Pharmacy, Clothing store, Restaurant/café/diner, Medical clinic, Convenient store, Government office, Farmers market, Child care facility, Social services center, Hardware, Museum	Checked Unchecked
Traffic safety	1. *There are bike lanes on most of the streets **	4 Pt. Likert Scale
	2. *There are sidewalks on most of the streets **	
	3. *Sidewalks are separated from road/traffic by parked cars **	
	4. *There is grass/dirt strip that separates the streets from most of the sidewalk **	
	5. *There are dirt trails on most of the streets **	
	6. *There are crosswalks and pedestrian signals to help walkers cross busy streets **	
	7. *The streets have speed bumps **	
	8. *The speed limit is 25 mph or less on most of the streets **	
Surveillance	1. *My neighborhood streets are well lit at night **	4 Pt. Likert Scale
	2. *Most units have front porches **	
	3. *The buildings are located close to the street **	
	4. *Most dwellings have front garage doors*	
	5. *My neighborhood has back alleys with garages **	
Experience	Aesthetics	4 Pt. Likert Scale
	1. *There is graffiti in my neighborhood*	
	2. *There is trash/litter in my neighborhood*	
	3. *There are many attractive natural sights to look at while walking **	
	4. *There are attractive buildings and homes*	
	5. *Possible interactions with wildlife makes it attractive to go on walks **	
	6. *Possible interactions with wildlife or stray dogs makes it unsafe to go on walks*	
	Slope	
	7. *Most streets are hilly making it difficult to walk or bike*	
	Way-finding	
	8. *It is easy to get lost while walking*	
	9. *There is clear signage or landmarks that help me find my way**	
	Thermal comfort	
	10. *There is enough shade to walk comfortably **	
	11. *There are trees along the streets **	
Greenspace	Proximity to Greenspace	
	1. *How far is the nearest greenspace from your home? **	¼ mile (5 min walk) 1/2 mile (10 min walk) Farther that 1/2 mile
	2. Greenspace is a *services close to home*	Checked Unchecked
	Access to Greenspace	
	3. *It is easy to walk to greenspace from my home **	4 Pt. Likert Scale
Community	1. Selecting/not-selecting “Community facilities” and “Church” as *services close to home*	Checked Unchecked
	2. *My neighborhood shares facilities (e.g., pool, tennis courts, community center) **	4 Pt. Likert Scale

* Questions that were reversed from their original source format in order to be consistent with intent of the instrument to capture increasing levels of walkability. Note: This questionnaire was based on previously validated tools [36,57], findings from previous studies [34], and design elements included in Leadership for Energy and Environmental Design for Neighborhood Development (LEED-ND) [28].

**Table A3:** Questions used to measure social interactions with neighbors.

Questions for Social Interactions with Neighbors	Options
1. *What activities do you or your family do in the streets of your neighborhood?* Option: Talk to neighbors	a. Checked b. Unchecked
2. *My neighborhood is friendly **	4 Pt. Likert Scale
3. *I greet some of my neighbors at least once a month **	
4. *In case of an emergency, I would ask a neighbor for help **	

* Questions that were reversed from their original source format in order to be consistent with intent of the instrument to capture increasing levels of walkability.
